# A reproducible number-based sizing method for pigment-grade titanium dioxide

**DOI:** 10.3762/bjnano.5.192

**Published:** 2014-10-21

**Authors:** Ralf Theissmann, Manfred Kluwig, Thomas Koch

**Affiliations:** 1 Research Services / Analytical Intelligence, KRONOS INTERNATIONAL, Inc., Peschstrasse 5, 51373 Leverkusen, Germany

**Keywords:** electron microscopy, particle size, pigment, sizing, titanium dioxide

## Abstract

A strong demand for reliable characterization methods of particulate materials is triggered by the prospect of forthcoming national and international regulations concerning the classification of nanomaterials. Scientific efforts towards standardized number-based sizing methods have so far been concentrated on model systems, such as spherical gold or silica nanoparticles. However, for industrial particulate materials, which are typically targets of regulatory efforts, characterisation is in most cases complicated by irregular particle shapes, broad size distributions and a strong tendency to agglomeration. Reliable sizing methods that overcome these obstacles, and are practical for industrial use, are still lacking. By using the example of titanium dioxide, this paper shows that both necessities are well met by the sophisticated counting algorithm presented here, which is based on the imaging of polished sections of embedded particles and subsequent automated image analysis. The data presented demonstrate that the typical difficulties of sizing processes are overcome by the proposed method of sample preparation and image analysis. In other words, a robust, reproducible and statistically reliable method is presented, which leads to a number-based size distribution of pigment-grade titanium dioxide, for example, and therefore allows reliable classification of this material according to forthcoming regulations.

## Introduction

Titanium dioxide is among the ten most abundant materials on the Earth [1]. In the form of a fine powder, it is used as white pigment in many application systems such as paints, plastics, paper and building materials. It is also used in cosmetics, foods and pharmaceuticals. Its superior properties as white pigment are based on its high refractive index, leading to maximum whiteness and opacity, if its particle size distribution is optimized for best scattering efficiency according to Mie's theory [2–3]. The most common industrial processing routes are the sulfate process and the chloride process. In the sulfate process, for example, ilmenite ore is dissolved in sulfuric acid, iron and titanium are separated by controlled precipitation, and colouring transition metals are removed in a bleaching process prior to calcination. In the chloride process, rutile in the form of sand or slag, for example, is treated with gaseous chlorine to form titanium tetrachloride. The titanium tetrachloride is purified by distillation and then transformed into solid TiO_2_ in a combustion process. After both processes, the resulting powders are de-agglomerated by standard milling procedures and then subjected to a finishing process, which is generally followed by a final de-agglomeration step. In the ideal case, the size (distribution) of the final products is optimised for the optical pigment properties, which are described by using Mie's theory. Mie's theory states that the optimum particle size for refracting light is just about half the wavelength it is meant to interact with. Speaking of visible light, a mean wavelength of 500 nm is reasonable to assume. Therefore, a mean pigment size of 250 nm is close to what the titanium dioxide makers are producing.

The calcination temperature, as the final step of the powder synthesis of the sulfate process, gives the choice of producing either the low-temperature crystallographic phase, anatase, or the high-temperature crystallographic phase, rutile. The chloride process leads to formation of rutile phase particles due to the high temperatures of the combustion process. Both phases have a number of advantages and disadvantages, which lead to their typical applications. Preferred fields of application for rutile pigments are coatings, paints, plastics and building materials, whereas anatase pigments are mainly used in cosmetics, pharmaceuticals or food. One of the most important properties of titanium dioxide is its UV absorption, which protects human skin against sunburn and skin cancer. Optically transparent TiO_2_ is the most important ingredient of any commercially available sun cream. The most common way of achieving a transparent, highly UV-absorbent sun cream is to use intentionally manufactured nano-sized titanium dioxide.

An urgent demand for reliable methods for the characterization of particulate materials is triggered by the prospect of forthcoming national and international regulations concerning the classification of nanomaterials [3–6]. Scientific efforts to establish standardized methods for determining number-based size distributions have so far been focused on model systems, such as spherical gold particles, spherical latex particles or spherical silica particles [7–10]. The typical obstacles in the characterization of industrial materials, such as irregular particle shapes [11], a broad size distribution [12] and strong aggregation and agglomeration effects [13–14], have not been addressed successfully. But as industrial materials are the goal of all regulatory efforts, robust, reproducible and reliable methods for the determination of the number-based size distributions for a wide range of industrial materials are urgently needed [3].

**Figure 1:**
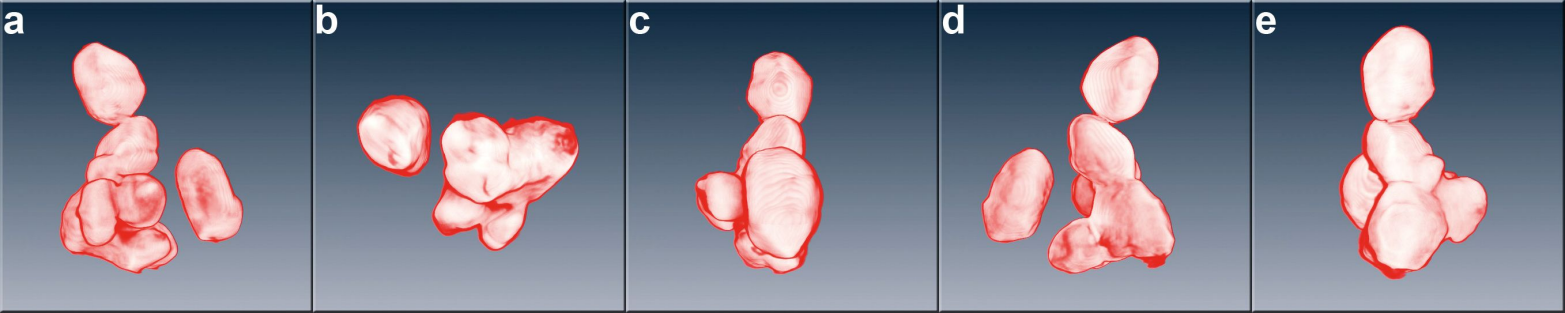
Schematic view of five different projections of one agglomerate found in KRONOS 2360, reproduced from an electron tomogram. The images (a) through (e) demonstrate that the projected area of the pigments varies greatly, depending on viewing direction. Moreover, they illustrate the difficulty with particle detection in projection images in the event of overlapping particles.

This study focuses on two commercially available pigments, KRONOS K2360 and KRONOS K1171. The former is a pigment with a rutile structure for use in coatings and paints, while the latter is a food-grade pigment with an anatase structure. We decided to develop a procedure that requires a minimum of subjective, user-based decisions. The major task to tackle on the way is to prepare a sample suitable for automated particle detection. Since automated detection routines are primarily based on grey-value thresholding, overlapping particles are a serious problem, which is illustrated in Figure 1. As an example, Figure 1 shows five different projections of one agglomerate found in K2360. The number of obviously visible primary particles varies for the different projections, as well as the size of the projected area, which is generally evaluated. These TEM tomogram-based data illustrate the fundamental problem associated with number-based particle size measurements: A true size distribution of the pigment will only be obtained if the full, three-dimensional shape of the particles is evaluated, e.g., as the equivalent sphere diameter or a minimum 3D Feret diameter. All known evaluation methods based on the evaluation of projections or slices contain systematic deviations [15–18]. As for every particle sizing method, besides the physical principles applied, the decision as to how to measure the particle size and its distribution is somewhat arbitrary and generally restricted by practicality aspects.

As the first selective decision, the present study uses polished sections in which overlapping particles are ruled out. As a consequence, the measurement used here does not correspond to a projected area of the particles, but to a section through the particles. The correlation between a measured size distribution and the "real" size distribution is the subject of so-called stereologic correction: Based on the assumption of a common, known particle shape, stereologic corrections may be used to estimate the "real" size distribution, either from the measured projection sizes or from the measured section sizes [15,19–20]. It is a fact that there is nothing like a common or known particle shape for titanium dioxide pigments. The presence of arbitrary shapes in industrially produced titanium dioxide makes stereologic correction impractical, both for the evaluation of projections and for the evaluation of sections. Consequently, the choice of the measuring technique is somewhat arbitrary. The technique presented in this study was chosen because of its high degree of automation and reproducibility. The effect of lacking stereologic correction, if significant, leads to a result which is slightly smaller than the "real" particle size. Therefore, the uncorrected section-size distributions presented in this publication give lower estimates of the real particle size distributions of the analysed pigments. For the intended purpose, i.e., classification of the material according to the recommendation of the European Commission [4], the method gives a conservative estimate of the particle size distribution.

## Results and Discussion

In order to establish the proposed method, especially for the sizing of pigment-grade titanium dioxide, the reproducibility of the method was primarily tested. Each pigment was prepared several times according to the procedure described in the experimental section. The first preparation run included four samples, labelled Ra01 through Ra04 for the rutile pigment and Aa01 trough Aa04 for the anatase pigment. A second preparation run was done several days later. It included the rutile samples Rb05 through Rb10 and the anatase samples Ab05 through Ab08. The first measurement of each sample is labelled M1; the second measurement was performed by a different operator and is labelled M2. The measured results in terms of minimum Feret diameter and equivalent circle diameter (ECD) are summarised in Tables 1 to 4. The first column gives the number of detected primary particles, followed by mean and standard deviation. Meaningful quantiles (d_10_, d_16_, d_25_, d_50_ and d_84_) are given in the next columns. The data of the different samples are treated without any assumption of a model for the distribution function. Consequently, no standard error based on the standard deviation and the number of particles counted is given. Instead, the standard error is calculated as the standard deviation of the repeated measurements, as done for the mean and all evaluated quantiles. The standard error is used to calculate the 95% confidence interval.

**Table 1:** Evaluation of the primary particle sizes of KRONOS K2360 in terms of the equivalent circle diameter (ECD); all values given in nm.

**ECD**	# particles	**mean**	std. dev.	d_10_	d_16_	d_25_	**d_50_**	d_84_

Ra01_M1	9763	**188.5**	58	114.1	130.4	149.3	**183.4**	246.5
Ra02_M1	9912	**187.5**	57.6	113.7	129.9	148.6	**182.3**	245.1
Ra03_M1	8753	**183.3**	56.3	111.2	127	145.3	**177.6**	239.5
Ra04_M1	9729	**189.2**	56.4	116.9	132.8	151.1	**183.7**	245.6
Rb05_M1	9056	**186.7**	57.4	113.1	129.3	148	**182.1**	244.1
*Rb05_M2*	*8684*	***188.9***	*57.9*	*114.7*	*131*	*149.8*	***181.7***	*246.8*
Rb06_M1	8430	**186.4**	58.2	111.8	128.2	147.1	**181.6**	244.6
*Rb06_M2*	*9629*	***186.6***	*58.1*	*112.2*	*128.6*	*147.5*	***181.7***	*244.7*
Rb07_M1	8362	**186.7**	57.3	113.2	129.4	148	**181.1**	244.1
Rb08_M1	6909	**187.3**	57.2	114	130.1	148.7	**182.9**	244.5
Rb09_M1	9013	**187.5**	58.5	112.6	129	148.1	**183.2**	246
Rb10_M1	9023	**185.0**	57.7	111	127.3	146.1	**180.9**	242.7

mean		**187.0**	**57.6**	**113.2**	**129.4**	**148.1**	**181.8**	**244.5**
std. error		**1.6**	**0.7**	**1.6**	**1.6**	**1.6**	**1.6**	**1.9**

confidence level (95%)	lower limit	**183.9**		110.1	126.3	145	**178.7**	240.8
	upper limit	**190.1**		116.3	132.5	151.2	**184.9**	248.2

**Table 2:** Evaluation of the primary particle sizes of KRONOS K2360 in terms of the minimum Feret diameter; all values given in nm.

**min. Feret**	# particles	**mean**	std. dev.	d_10_	d_16_	d_25_	**d_50_**	d_84_

Ra01_M1	9763	**171.8**	53.2	103.7	118.7	136	**166.2**	225
Ra02_M1	9912	**171.2**	52.7	103.6	118.4	135.6	**166.2**	223.9
Ra03_M1	8753	**167.3**	51.7	101.1	115.7	132.5	**161.9**	219
Ra04_M1	9729	**172.9**	51.6	106.8	121.3	138.1	**167.9**	224.4
Rb05_M1	9056	**170.2**	52.6	102.7	117.5	134.7	**166**	222.8
*Rb05_M2*	*8684*	***172.1***	*53.3*	*103.8*	*118.8*	*136.2*	***166.1***	*225.3*
Rb06_M1	8430	**169.7**	53.3	101.4	116.4	133.8	**165.1**	222.9
*Rb06_M2*	*9629*	***170.7***	*53.3*	*102.4*	*117.4*	*134.7*	***166.2***	*224*
Rb07_M1	8362	**170.2**	52.5	102.9	117.7	134.8	**165.3**	222.6
Rb08_M1	6909	**170.5**	52.4	103.4	118.2	135.2	**166.2**	222.9
Rb09_M1	9013	**170.8**	53.5	102.2	117.3	134.7	**166.2**	224.4
Rb10_M1	9023	**168.6**	52.9	100.8	115.7	132.9	**163.7**	221.5

mean		**170.5**	**52.7**	**102.9**	**117.7**	**134.9**	**165.6**	**223.2**
std. error		**1.5**	**0.6**	**1.6**	**1.5**	**1.5**	**1.5**	**1.7**

confidencelevel (95%)	lower limit	**167.6**		99.8	114.8	132	**162.7**	219.9
	upper limit	**173.4**		106	120.6	137.8	**168.5**	226.5

**Table 3:** Evaluation of the primary particle sizes of KRONOS K1171 in terms of the equivalent circle diameter (ECD); all values given in nm.

**ECD**	# particles	**mean**	std. dev.	d_10_	d_16_	d_25_	**d_50_**	d_84_

Aa01_M1	5766	**152.2**	49.6	88.5	102.5	118.7	**146.4**	201.8
*Ab01_M2*	*6073*	***151.8***	*49*	*89.1*	*102.9*	*118.8*	***146.7***	*200.8*
Aa02_M1	6205	**150.6**	47.6	89.5	102.9	118.4	**144.4**	198.2
*Ab02_M2*	*5743*	***153.5***	*49.2*	*90.4*	*104.3*	*120.3*	***147.8***	*202.7*
Aa03_M1	5906	**152.7**	48.6	90.4	104.1	119.9	**146.3**	201.3
Aa04_M1	6280	**150.2**	47.9	88.8	102.3	117.9	**144**	198.1
Ab05_M1	5250	**149.9**	50.1	85.7	99.8	116.1	**144.6**	200.1
Ab06_M1	5565	**152.7**	49	89.9	103.7	119.7	**147**	201.8
Ab07_M1	5808	**150.2**	49	87.4	101.2	117.2	**144.2**	199.2
Ab08_M1	5901	**151.6**	48.7	89.2	102.9	118.8	**146.1**	200.4

mean		**151.5**	**48.9**	**88.9**	**102.7**	**118.6**	**145.7**	**200.4**
std. error		**1.3**	**0.7**	**1.4**	**1.3**	**1.3**	**1.3**	**1.6**

confidence level (95%)	lower limit	**149.1**		86.1	100	116.1	**143.1**	197.4
	upper limit	**154**		91.7	105.3	121	**148.4**	203.5

**Table 4:** Evaluation of the primary particle sizes of KRONOS K1171 in terms of the minimum Feret diameter; all values given in nm.

**min. Feret**	# particles	**mean**	std. dev.	d_10_	d_16_	d_25_	**d_50_**	d_84_

Aa01_M1	5766	**139.2**	45.3	81.2	93.9	108.7	**133.3**	184.6
*Ab01_M2*	*6073*	***138.9***	*44.6*	*81.7*	*94.2*	*108.8*	***133.7***	*183.5*
Aa02_M1	6205	**137.9**	43.6	82	94.2	108.5	**132.6**	181.5
*Ab02_M2*	*5743*	***140.4***	*45*	*82.8*	*95.4*	*110.1*	***135.1***	*185.4*
Aa03_M1	5906	**139.5**	44.4	82.6	95.1	109.6	**133.7**	183.9
Aa04_M1	6280	**137.7**	43.8	81.6	93.9	108.2	**132.4**	181.5
Ab05_M1	5250	**137.5**	45.6	79	91.8	106.7	**132.5**	183.1
Ab06_M1	5565	**139.9**	44.8	82.4	95	109.6	**133.9**	184.7
Ab07_M1	5808	**137.9**	44.8	80.4	93	107.6	**132.5**	182.7
Ab08_M1	5901	**139**	44.4	82.1	94.6	109.1	**133.7**	183.4

mean		**138.8**	**44.6**	**81.6**	**94.1**	**108.7**	**133.3**	**183.4**
std. error		**1.0**	**0.6**	**1.2**	**1.1**	**1.0**	**0.8**	**1.3**

Confidence level (95%)	lower limit	**136.8**		79.3	92	106.7	**131.7**	180.9
	upper limit	**140.8**		83.8	96.2	110.6	**135**	186

The high reproducibility of the measurement, which leads to a standard error below 2 nm for all evaluated measurements, justifies use of the non-parametric rank sum test of Siegel and Tukey [21] to test whether the measured distribution can be attributed to the same population. The comparison of all measured samples proves that this is the case, both for KRONOS K2360 and for KRONOS K1171. No significant differences between the standard errors for the ECD and minimum Feret diameter measurements are obtained for the rutile pigment. For the anatase pigment, lower standard errors are found for the minimum Feret diameter. This is attributed to a less regular shape as a consequence of the production process.

The data prove that the method presented for primary particle sizing of pigmentary titanium dioxide is highly robust and reproducible. This is the consequence of a rigorous procedure, which primarily targets reliability: a representative, macroscopic amount of sample to start with, preparation that eliminates the possibility of overlapping particles, defined standard operating procedures for sample preparation, measurement and evaluation, and a minimum influence of operator-based uncertainties. The result is an obtained standard error of less than 2 nm and a relative coefficient of variation below 1.6% for all measured quantities.

However, the method has several limitations, which also need to be addressed and discussed here. First of all, the procedure was developed for pigment-grade titanium dioxide prepared as a cross-section. An adjustment of the method may possibly be needed for its application to other particulate materials with other size distributions. The presented results are, accurately speaking, the distribution of ECD and minimum Feret diameter of the pigment sections. Stereologic correction has not been performed, and is not feasible for this application [15,20]. Errors introduced by stereologic effects may gain importance if the size of the measured objects is large compared to the penetration depth of the electron beam. The size distributions measured from polished sections can be expected to differ from those of projected areas. However, the high reproducibility of the proposed method will certainly allow comparison with any other sizing method, as long as a reference or standard for this purpose is present.

## Conclusion

A highly reproducible, statistically tested method for the sizing of pigment-grade rutile and anatase is established. The standard error of the method is shown to be below 2 nm for all measured quantities, the relative coefficient of variation being below 1.6%. The presence of a systematic bias due to the lack of stereologic correction cannot be verified for the time being, since a certified standard for titanium dioxide is still lacking. The reproducibility of the method is based on the use of a representative, macroscopic amount of sample material, a high degree of automation, the elimination of detection errors due to overlapping particles and a transparent filtering procedure for detected particles. As a consequence, the method presented is well suited to classifying pigment-grade titanium dioxide according to the recommendation of the EU Commission of 18 October 2011 on the definition of particulate nanomaterial, or for other forthcoming regulations in the future.

## Experimental

The experimental procedures described below are in full agreement with the practical guide for particle size characterization published by NIST [22].

### Sample preparation

A well-prepared sample is the basis for reliable evaluation of the size distribution of a pigment. The limited quantity of samples used in electron microscopy emphasises this point. Starting with approximately 10 g pigment, which is assumed to be a representative sample, the following steps are taken:

1. The "cone and quartering" method is repeatedly used to divide the sample in half until a quantity of 2 g is left.
2. The 2 g of pigment are thoroughly mixed with 4 g hot-mounting resin and 22.5 g ZrO_2_ milling beads. Mixing is done for 4 min at a moderate frequency of 20 Hz, using a Retsch Mixer Mill MM400. The milling beads are removed after the mixing process.
3. The mixture is then hot-mounted by using a Struers Citopress under standard conditions of 180 °C and 3 bar pressure.
4. A five-step polishing process, with active oxide polishing as the final step, leads to a smooth, scratch-free section through the embedded pigments.
5. As the final step before SEM investigation, an 8 nm Au/Pd conductive layer is deposited on top of the sample surface.

The sample preparation procedure summarised above leads to a sample with a high pigment density and allows imaging of several thousand pigments within a set of approx. 50 images. The "cone and quartering" method ensures that the embedded sample is representative. A macroscopic amount is taken for the mixing and embedding procedure. Since the whole representative amount is embedded, effects of flushing are ruled out and the low viscosity of the resin ensures that sedimentation is not a problem. The section finally analysed can therefore be reasonably assumed to be representative. The high area density of the pigment in all images ensures that constant conditions for the automated post-processing and detection procedure are assured.

### Measurement, pigment detection and size analysis

A working distance of 7 mm and an acceleration voltage of 5 kV with an Everhart–Thornley detector are chosen as the standard imaging conditions for SEM imaging. The microscope used is a Leo 1530VP SEM. A magnification of 20.000× (5.7 nm/pix) is chosen for the rutile sample, which displays a larger primary particle size, and a magnification of 30.000× (3.8 nm/pix) for the anatase sample, which has a significantly smaller primary particle size. In both cases, this corresponds to approx. 20 pixels for the equivalent circle diameter (ECD) of the 10% quantile d_10_ [23]. A silicon wafer with etched structures is used for calibration of the instrument. The size of the structures is certified by the PTB under Serial No. IMS-HR 08 3641-01 490 and the calibration mark 44049 11 PTB. An automated protocol (macro) for image post-processing and particle detection was developed. The procedure was realised with the commercial software package "analysis" from Olympus-SIS. All filters used are standard filters implemented in the software. The detailed settings are defined in the standard operating procedure and are listed below. This allows maximum transparency of the procedure and maximum comparability and reproducibility of the results, e.g., when dealing with regulatory authorities or their contracting measuring partners.

**Figure 2:**
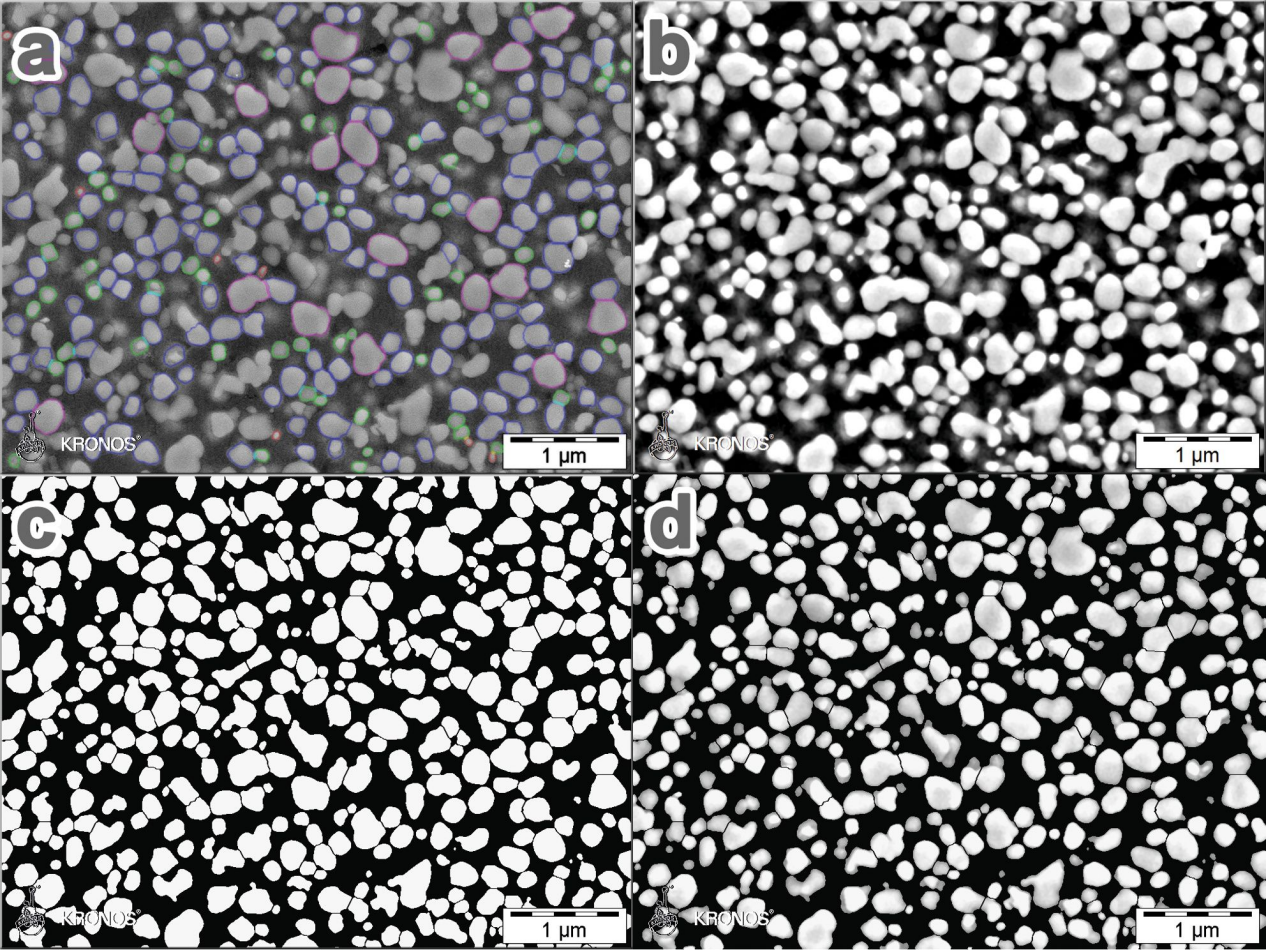
Imaging conditions and image post-processing for pigment sizing for a rutile pigment; a) original image superimposed with the outlines of the finally evaluated particle sections after grey-value and shape filtering; b) unified image after noise reduction and shading correction; c) binarised image after application of a watershed transform and connectivity filter; d) filtered image (b) masked with binarised image (c); automated detection applied to image d.

The full procedure includes unification of the grey values, masking of the unified image, particle detection and filtering of the results, as shown in the images of Figure 2. The first step ensures comparability, not only within the set of images of one sample, but also between different samples or even between samples measured on different instruments with varying detector settings or noise levels. The comparability is sufficient, as long as the penetration depth of the electrons, which is determined by the acceleration voltage, remains constant. In the second step, the images are binarised by using automated grey-value threshold determination [22]. A morphological particle separation filter, the watershed transform, is applied to the binary image. The resulting binary image with the separated particles is used as a mask for the unified image. The final detection of the primary particles is done on the masked unified image, which allows for the detection of the particles, including their grey values. The grey values of the particles are important for the subsequent filtering of the results obtained. It is used in order to remove particles that are located below the polished surface, but give rise to an increased intensity compared to the background. In general, these falsely detected particles show the lowest detected intensities and, therefore, also the lowest standard deviations. A combination of both values has proven adequate for grey-value filtering. Additional filtering is based on the pigment shape. As for the grey values, combined filtering of two shape characteristics has proven advantages, namely the so-called convexity and the shape factor:

**1. Unifying the grey values as the basis for final pigment detection** (Figure 2b)

- automated contrast and brightness adjustment
- median filter for noise suppression
- rank filter for noise suppression with a size of 5 pixels and a rank of 50%
- multiplicative shading correction for a size of 255 × 255 pixels and a cut-off level of 10%

**2. Preparing and applying a mask image, followed by particle detection** (Figure 2c,d)

- automated grey-value thresholding and binarisation
- repeated removal of pixels connecting only to three or less neighbouring pixels, using the connectivity filter
- applying the watershed transform in order to separate aggregated or adjacent particles (Figure 2c)
- pixel-by-pixel multiplication of the unified image with the mask image (Figure 2d)
- automated grey-value thresholding and particle detection

**3. Morphological and grey-value filtering of the detected particles to extract the primary particles** (Figure 2a)

- removing 10% of the particles with the lowest mean grey value of all detected particles
- removing 10% of the particles with the lowest standard deviation of the grey value of all detected particles
- removing all particles with a "shape-factor" below 0.86 as non-primary particles
- removing all particles with a convexity of less than 0.90 as non-primary particles

**Figure 3:**
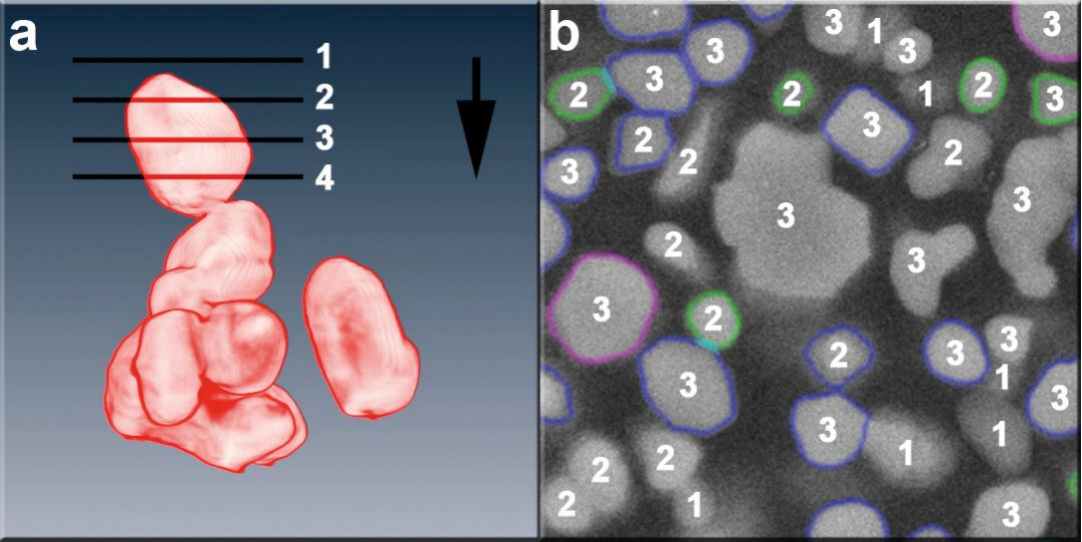
Visualisation of the systematic challenges in the detection of sectioned particles; a) principal possibilities for sectioning a particle; the viewing direction is indicated by the black arrow; lines 1 to 4 indicate section planes through the particle shown; b) a representative electron micrograph of a polished section; the numbers given correspond to the sections given exemplarily in part (a); the particles with a coloured envelope are the ones finally detected after grey-value and morphologic filtering.

Figure 3 visualises the particle detection characteristics that are intrinsic to the analysis of a polished section. The section through the particles can in principle be located close to the centre of mass, in which case the section gives the maximum area along the viewing direction for a convex particle (plane 3 in Figure 3a). But it can also be directed above the particle (plane 1 and 2 in Figure 3a) or below the centre of mass with respect to the viewing direction (plane 4 in Figure 3a), which leads to a smaller detected area. The cases of planes 3 and 4 in Figure 3 are essentially indistinguishable – they provide sharp particle edges. The cases of planes 1 and 2 give rise to a rim of lower intensities at the particle edges. Figure 3b shows how the different cases look in the SEM image, the numbers given corresponding to the section planes in Figure 3a. Only the particles with a coloured boundary are the ones finally detected after applying all filters. It can be seen that grey-value filtering effectively filters those particles that are located fully below section plane 1. Particles sectioned above the centre of mass, labelled with a 2, are only partially filtered out. The boundaries are detected reasonably well for the counted particles. The detection of particles sectioned close to the centre of mass or below the centre of mass is precise, both cases are labelled with a 3 in Figure 3b, since they are indistinguishable in the SEM image. Uncounted particles labelled with a 3 are filtered out as non-primary particles due to their shape.

## References

[1] Wedepohl K H (1995). Geochim Cosmochim Acta.

[2] Mie G (1908). Ann Phys.

[3] Theissmann R, Kluwig M, Koch T (2013). A reliable method for sizing constituent particles in pigment-grade titanium dioxide. Proceedings of the Microscopy Conference.

[4] European Commision (2011). Off J Eur Communities: Legis.

[5] Auffan M, Rose J, Bottero J-Y, Lowry G V, Jolivet J-P, Wiesner M R (2009). Nat Nanotechnol.

[6] Brown S C, Boyko V, Meyers G, Voetz M, Wohlleben W (2013). Environ Health Perspect.

[7] Klein T, Buhr E, Johnsen K-P, Frase C G (2011). Meas Sci Technol.

[8] Yoshida H, Mori Y, Masuda H, Yamamoto T (2009). Adv Powder Technol.

[9] Stubbs J M, Sundberg D C (2005). Polymer.

[10] Mori Y, Yoshida H, Masuda H (2007). Part Part Syst Charact.

[11] Naito M, Hayakawa O, Nakahira K, Mori H, Tsubaki J (1998). Powder Technol.

[12] Ferraris C F, Hackley V A, Avilés A I (2004). Cem, Concr, Aggregates.

[13] Hayakawa O, Nakahira K, Naito M, Tsubaki J (1998). Powder Technol.

[14] Linsinger T, Roebben G, Gilliland D (2012). Requirements on measurements for the implementation of the European Commission definition of the term 'nanomaterial'.

[15] Kötzer S (2006). Image Anal Stereol.

[16] Sahagian D L, Proussevitch A A (1998). J Volcanol Geotherm Res.

[17] Exner H E (2004). Image Anal Stereol.

[18] Wejrzanowski T, Lewandowska M, Kurzydłowski K J (2010). Image Anal Stereol.

[19] Hobolth A, Jensen E B V (2002). Image Anal Stereol.

[20] Payton E J (2012). J Miner Mater Charact Eng.

[21] Sachs L (1984). Angewandte Statistik.

[22] Jillavenkatesa A, Lum L-S H, Dapkunas S (2001). NIST Recommended Practice Guide: Particle Size Characterization.

[23] Wedd M (2010). Part Part Syst Charact.

